# Factors affecting delayed and non-receipt of healthcare during the COVID-19 pandemic for women in rural Maharashtra, India: Evidence from a cross-sectional study

**DOI:** 10.1016/j.eclinm.2022.101741

**Published:** 2022-11-16

**Authors:** Arnab K. Dey, Nandita Bhan, Namratha Rao, Mohan Ghule, Sangeeta Chatterji, Anita Raj

**Affiliations:** a Center on Gender Equity and Health, School of Medicine, University of California San Diego, La Jolla, CA, USA; b School of Social and Political Science, University of Edinburgh, United Kingdom; c Division of Social Sciences, Department of Education Studies, University of California San Diego, La Jolla, CA, USA

**Keywords:** COVID-19, Maternal and Child Health, Healthcare utilisation, Delay in seeking healthcare, Health system perception, Mediation, India

## Abstract

**Background:**

Pathways to low healthcare utilisation under the COVID-19 pandemic are not well understood. This study aims to understand women's concerns about the health system's priorities and their increased burden of domestic responsibilities during COVID-19 as predictors of delayed or non-receipt of needed care for themselves or their children.

**Methods:**

We surveyed married women in rural Maharashtra, India (N = 1021) on their health and economic concerns between Feb 1 and March 26, 2021. This study period was when India emerged from the first wave of the pandemic, which had severely impacted the health systems, and before the second—even more devastating wave had started. We captured if women were concerned about access to non-COVID health services due to healthcare being directed solely to COVID-19) (exposure 1) and whether their domestic responsibilities increased during the pandemic (exposure 2). Our outcomes included women's reports on whether they delayed healthcare seeking (secondary outcome and mediator) and whether they received healthcare for themselves or their children when needed (primary outcome). We conducted adjusted regression models on our predictor variables with each outcome and assessed the mediation effects of delayed healthcare seeking for each of the exposure variables.

**Findings:**

We found that women who were concerned that healthcare was directed solely towards COVID-19 were more likely not to receive healthcare when needed (Adjusted Risk Ratio [ARR] = 1.49, 95% CI = 1.14, 1.95). We also found that women whose domestic care burden increased under the pandemic were more likely to delay healthcare seeking (ARR = 1.84, 95% CI = 1.05, 3.21). Delayed healthcare seeking mediated the associations between each of our exposure variables with our outcome variable, non-receipt of needed healthcare.

**Interpretation:**

Our findings suggested that women's perceptions of healthcare systems and their domestic labour burdens affected healthcare seeking during the pandemic in India, even before the second wave of COVID-19 incapacitated the health system. Support for women and health systems is needed to ensure healthcare uptake during crises.

**Funding:**

Eunice Kennedy Shriver National Institute of Child Health and Human Development, National Institutes of Health, USA (grant numbers: R01HD084453- 01A1 and RO1HD61115); Department of Biotechnology, Government of India (grant #BT/IN/US/01/BD/2010); the EMERGE project (Bill and Melinda Gates Foundation Grants: OPP1163682 and INV018007; PI Anita Raj), and Bill and Melinda Gates Foundation Grant number INV-002967.

**Research in context**

**Evidence before this study**

On June 25, 2022, we searched PubMed and Google Scholar to review the literature on non-COVID healthcare-seeking behaviours during the pandemic, using the search terms “healthcare seeking” OR “healthcare utilization” OR “maternal and child healthcare” AND “COVID-19” OR “pandemic.” We reviewed only those papers published from Feb 1, 2020 to May 31, 2022, which included primary research data from LMIC contexts and focused on non-COVID-19 healthcare use. We found a reduction in non-COVID healthcare utilisation for maternal and child health services in several countries, but data from India were limited. Little research assessed what pathways led to this observed effect, motivating our consideration of women's perceptions of healthcare disruption, and increased domestic burdens as predictors of healthcare utilisation.

**Added value of this study**

Our study is set in a COVID-19 hotspot in India, at a time when the country was emerging from the first wave of the pandemic that had already strained the health system and before the second wave had begun, which would soon bring the country's health system to the brink of collapse. In our sample of rural women, one in three reported non-COVID-related healthcare needs for themselves or their children. Our study found that women's perception of an incapacitated health system and increased domestic care burden were associated with the non-receipt of healthcare when needed. We further found that delayed healthcare seeking mediated both observed associations, explaining 11% and 28% of the total effect of our two exposures on non-receipt of healthcare, respectively.

**Implications of all the available evidence**

Findings from the study emphasise the importance of greater outreach and communication by health services in times of crisis (e.g., outbreaks, natural disasters) to alleviate community concerns regarding the availability of health services. In conjunction, our findings also underscore the need to alleviate women's domestic labour burdens to reduce delayed healthcare seeking and non-receipt of needed care for women and children. While findings are specific to COVID-19, they likely reflect needs for women and children beyond the current context.

## Introduction

India has been severely affected by the COVID-19 pandemic in terms of infections and deaths, having faced multiple severe outbreak waves.^1^ The first wave of the pandemic in India saw a peak in September 2020 that gradually declined by the end of January 2021. While this wave had put some strain on the country's health system, the second wave of the pandemic that began in March 2021 was particularly devastating, bringing the health system almost to the brink of collapse by the summer of 2021.^2^^,^^3^ Globally, the pandemic caused unprecedented shortages in medical supplies and healthcare workers,^4^^,^^5^ compromising non-COVID-related healthcare and preventative medicine. Consequently, non-COVID-19 health concerns have also increased, especially for women and children, due to delayed or non-receipt of healthcare.^6^, ^7^, ^8^, ^9^, ^10^ Modelling estimates suggest that even with conservative assumptions, the COVID-19 pandemic will result in 253,500 additional child deaths and 12,200 additional maternal deaths,^11^ primarily due to health system disruption and non-receipt of care.^12^, ^13^, ^14^, ^15^ In some contexts, including in India, non-COVID-19 care was unavailable in some clinical settings.^4^^,^^16^

However, what remains less clear is the loss of care attributable to women not obtaining care for themselves and their children due to worries about the health system's capacities under the pandemic. Studies from clinic-based populations or contexts outside of India have shown that women delayed or avoided accessing non-COVID-related services for themselves or their children due to reduced mobility during the lockdowns, fear of becoming infected in a clinical setting, and a perception that the health system will not be able to cater to the needs of non-COVID patients due to a strain on its resources.^16^, ^17^, ^18^ This is of particular concern in low- and middle-income settings, which have seen pandemic-related reductions in the uptake of HIV and family planning services,^19^, ^20^, ^21^, ^22^ antenatal care^23^^,^^24^ and other maternal health services,^9^^,^^10^ and preventive care for children such as immunisation.^8^^,^^9^^,^^11^^,^^25^, ^26^, ^27^

Increased household burdens for women under the pandemic have also been documented in India and elsewhere.^28^^,^^29^ These too may affect women's ability to pursue care, particularly in rural contexts where healthcare services are often farther from home. To date, studies have not examined the attributes that affect pathways to women's lower utilisation of healthcare services during the COVID-19 pandemic, such as concerns regarding health system capacities and increased domestic labour burdens. Such an understanding is crucial to restoring non-COVID health service provision and utilisation once the catastrophic effects of the pandemic start to subside. Findings can also guide responses to future pandemics and other crises.

This study examines the associations of a) women's concern about health system capacities and b) their increased domestic responsibilities under the pandemic with the outcomes of delayed and non-receipt of health care in a COVID-19 hotspot in rural India. Our primary hypothesis is that women's perception of the health system's focus on non-COVID care (exposure-1) and increased burdens of domestic responsibilities (exposure-2) will be associated with the non-receipt of needed health care (primary outcome) and delayed healthcare seeking (secondary outcome) for themselves or their child during the pandemic. We also hypothesise that delayed healthcare seeking will mediate the association between our exposure variables and our primary outcome, the non-receipt of needed healthcare.

## Methods

### Study setting and sample

The study was conducted in rural Maharashtra, in Pune District, a region that experienced a higher COVID-19 burden in 2020 and 2021.^30^ We collected data in early 2021, at a time when the country was emerging from the effects of the first wave of the pandemic and before the second, and more devastating wave had begun to take its toll on the country's health system.

Our sample comprised a cohort of women who participated in a family planning intervention trial^31^ and had agreed to participate in other studies. The sample for the parent study was drawn from the catchment of 5 Primary Health Centres (PHCs) in Junnar Taluka, in the rural Pune district. These PHCs were identified in consultation with officials from the department of health and family welfare in Maharashtra and from district health offices in Pune. The 5 PHCs included 20 sub-centres (the most local facilities in India's public health system) and catered to a population of about 30,000 households spread across 41 villages and hamlets. The parent study involved n = 60 eligible couples from each of the 20 sub-centres. Couples were eligible for the parent study if women were in the age range of 18–30 years, Marathi speaking, and residing in the village of recruitment for the past three months.^31^ Couples were excluded if either of them was cognitively impaired or sterilised. The latter criterion was used because the parent study focused on family planning. The field research team for the parent study first conducted household screening in each of the twenty sub-centres and created a list of eligible couples residing in the catchment area of the sub-centre. Subsequently, n = 60 couples were randomly selected from the list from each of the 20 sub-centres for inclusion in the parent study. This led to a total sample size of N = 1201 with an additional couple recruited than intended due to simultaneous recruitment. All participants provided informed consent before screening and had also agreed to participate in future studies. Further details about the sampling procedure are described in the study protocol for the trial.^31^

**Fig. 1:**
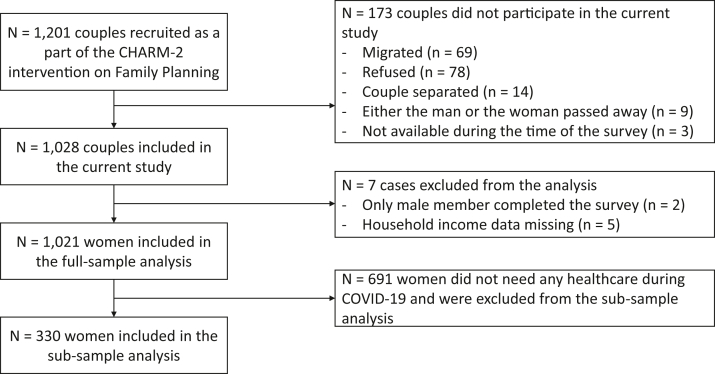
**Flowchart depicting the inclusion of participants inthe study.**

For the present study, we followed up with all the couples included in the parent study between Feb 1 and March 26, 2021. Of the 1201 couples included in the trial, 173 did not participate in the current study due to migration (n = 69), refusal to participate (n = 78), separation/divorce (n = 14), death of either one or both partners (n = 9), and non-availability (n = 3). Of the remaining 1028 couples, 7 cases were further excluded due to missing information on key variables. This left a total of 1021 couples for inclusion in the current study, with a participation rate of 85 percent relative to the parent study. This study focuses on data collected from women in each household. Fig. 1 depicts a flowchart of the inclusion of participants in this study.

### Data collection

Trained enumerators reached out to participants who had agreed to be contacted for future studies and invited them into this study on the effects of COVID-19 on their household financial and health situation. Participants provided written consent before participation. We engaged in mandated COVID-19 safety protocols for all data collection. All participants and enumerators wore masks during the survey, and all surveys were conducted outdoors. Temperature measurements were taken before enumerators started working. No documented cases of infection or transmission occurred as a consequence of study participation for our participants or enumerators. We collected all data with electronic tablets, and data were uploaded automatically into a database once every day. We did not include any identifiable data in the survey but had a unique identifier to link data collected in this study to previous rounds of data collection for sociodemographic data.

### Ethical approval

The institutional review boards of the University of California San Diego, the Indian Council of Medical Research, and the Population Council approved the parent study. All procedures for the current study were reviewed and approved by the Institutional Review Board of the University of California at San Diego (Project Number 202032XX) and by Sigma-IRB (IRB Number 10037/IRB/20-21).

### Measures

#### Exposure variables

We had two exposure variables of interest. The first (exposure 1) was women's concern that the health system solely focuses on COVID-19 and no other health concerns. Women were asked: “Are you worried that health care is only being directed to COVID-19 and not other concerns?” Responses were categorised as “yes” (coded as ‘1') or “no” (coded as ‘0'). Our second exposure variable (exposure 2) was self-reports of the increased burden of women's domestic responsibilities, as this too may impede women's ability to get care for themselves and their children. Women were asked a series of four items that started with the following statement: “For each of the following activities, please tell me about how many hours a day you typically spend on the activity and whether this is more or less time than you spent on this activity before the COVID-19 pandemic.” This statement was followed by the following four items: a) Getting health services for self or other family members, b) Cooking for the family, c) Other domestic work (including cleaning, fetching wood or water), and d) Unpaid care for children, ill family members, or the elderly. Women who reported that their responsibilities “increased” for each of these items were coded as ‘1’ and those who responded that their responsibilities for these items had “decreased” or “stayed the same” were coded as ‘0’. We then added these four items to create an ordinal variable for our second exposure. This ordinal variable ranged from 0 to 4, with a value of 0 indicating that women's responsibility had not increased for any of the four items listed above and values of 1 through 4 indicating that their responsibility had increased in 1, 2, 3, or 4 household activities, respectively.

#### Outcome variables

We considered two outcome variables in this study: delayed healthcare seeking (secondary outcome and potential mediator) and non-receipt of care when needed (primary outcome). To assess these variables, we first identified women who needed healthcare for themselves or for their children during the pandemic. We asked women if they needed healthcare (not related to COVID-19) for themselves or their children during the pandemic. We captured the need for healthcare via a single item, impeding our ability to disentangle whether the respondent or the child (or both) needed care. A total of n = 330 women reported that they needed non-COVID-related healthcare for themselves or for their children during the pandemic, and we used this sub-sample to create our outcome variables.

Our primary outcome was the non-receipt of healthcare for self or child when needed during the pandemic. We asked women reporting the need for care: 1) “Did the COVID-19 pandemic ever result in you not getting health care when you needed it?”; and 2) “Did the COVID-19 pandemic ever result in you not getting health care for your child/children when they needed it?”. Women who reported that either they or their child did not get the healthcare when needed were coded as ‘1’. Women who said that they and their children got healthcare when needed were coded as ‘0'.

The secondary outcome (and potential mediating variable) was delayed healthcare seeking. We asked women the following question: “Have you delayed or not pursued any of the following types of care for yourself or your children when you needed it due to the COVID-19 pandemic?” The response options included 1) Family planning or reproductive health care; 2) Maternal or antenatal care 3) Institutional delivery; 4) Neonatal care for my new-born; 5) Vaccinations for my children; 6) Health care when my child was sick with fever or infection; 7) Health care when I was sick with fever or infection; 8) No, this has not been a concern. Participants were asked to select all response options that applied to them. Delay in seeking healthcare was considered as a dichotomous variable as opposed to an ordinal variable using the eight response options described above. This choice was made because treating the ‘delay in seeking healthcare' variable as an ordinal variable, with eight response options would have made the individual cell sizes of each category to be very small, thereby precluding us from any meaningful analyses. The dichotomous variable was constructed by coding all women who reported that they delayed seeking care (i.e., selected any response options 1–7) as ‘1' and all other women as ‘0'.

#### Covariates

Our covariates in the model included socio-demographics and COVID-19 effects. For that, we used women's and their partner's ages, years of education, women's religion, caste, parity, and household income. We treated women's and men's age, years of education, and household incomes as continuous variables in the models. We used religion as a dichotomous variable and coded it ‘1' if women reported following Hinduism and ‘0' if they followed other religions. We used caste as a categorical variable with four categories: a) Scheduled Caste (SC); b) Scheduled Tribe (ST); c) Other backward Classes (OBC), and d) General/Other. We coded couples who did not have children as ‘0', those with a single child as ‘1', and those with two or more children as ‘2'.

For COVID data, we included variables on any COVID-19 infections in the household and any incidents of women's reports of economic hardship during the pandemic as covariates in the models. For each member of the household, respondents were asked if that household member had reported any fever/cough or had tested positive for COVID-19 30 days before the survey date. We created a dichotomous variable to capture any COVID-19 infections, coded ‘1' if any member in the household had a fever/cough or had tested positive for COVID-19 30 days before the survey and '0' otherwise. We created a dichotomous variable to capture economic hardship and coded it ‘1' if women reported that they faced economic hardship during the pandemic and ‘0' otherwise.

### Statistical analysis

We undertook univariate analyses for all the variables used in the study (mean and standard deviations for continuous variables and frequency and percentage for categorical variables) for the full sample and for the subsamples of those who needed non-COVID healthcare for themselves or their child during the pandemic and those who did not need care.

Subsequently, we developed three separate Poisson regression models to study the associations between our exposure variables and outcomes for the subsample of those requiring healthcare for themselves or their children during the pandemic. First, we conducted an adjusted regression model to assess the effect of each exposure variable on our primary outcome, non-receipt of care for self or child when needed (Model 1). Second, we developed a model similar to Model 1 and additionally adjusted for the delay in seeking healthcare (Model 2). Third, we conducted an adjusted regression model to estimate the effects of each exposure variable on delayed healthcare seeking (Model 3). Since Poisson regression is known to over-estimate the error for the risk ratios when applied to binomial data, we use calculated robust confidence intervals using sandwich estimators for all our models.^32^^,^^33^

Given a large number of covariates and the relatively small sample size, we used the backward selection method to identify covariates to be included. This process identified *any COVID-19 infections in the household* and *women's education* for inclusion. We retained these variables as covariates and additionally included women's age and parity as covariates since these variables were potential confounders in our exposure-outcome association.

#### Causal mediation analysis

**Fig. 2:**
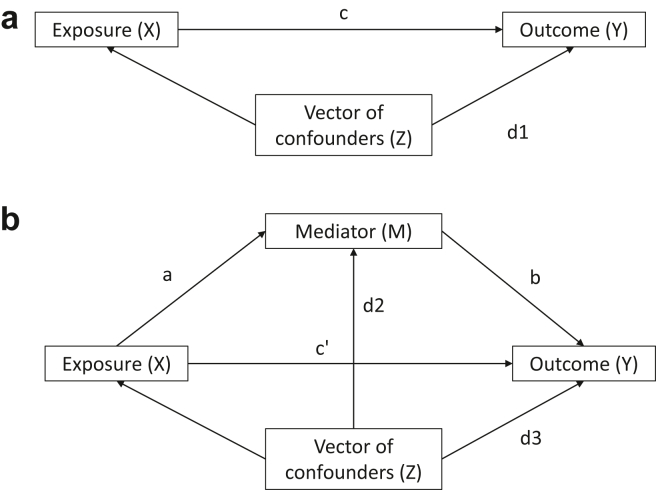
**Illustration of a model with one mediator and a vector of confounders.** a: path c captures the total effect of the exposure (X) on the outcome (Y). Z is a vector of confounders between X and Y. b: path c' captures the direct effect of the exposure (X) on the outcome (Y), and paths a and b quantify the indirect effect of the exposure on the outcome via the mediator (M). Z is a vector of confounders between X and Y.

Finally, we performed causal mediation analyses to examine whether delayed healthcare seeking mediated the association between our exposures and primary outcome. Causal mediation analysis decomposes the total effect of an exposure on an outcome into natural direct effects and natural indirect effects. The natural direct effects quantify the effect of the exposure on the outcome by blocking the pathway via any mediator. The natural indirect effects capture the effect of the exposure on the outcome transmitted via the mediator by holding the exposure constant.^34^ Fig. 2 presents a schematic for a simple mediation model, where path c in Fig. 2a captures the total effect of the exposure on the outcome, path c' in Fig. 2b captures the direct effect of the exposure on the outcome, and paths a and b quantify the indirect effect of the exposure on the outcome via the mediator.

When the mediator (M) and outcome (Y) are both binary, and the outcome is rare (<10%), we can estimate the direct and indirect effects of exposure (X) using the following three logistic regression equations:

(1) $$logit\left(\mathrm{Pr}(Y=1|X,Z\right)=i_{y1}+cX+d_{1}Z$$

(2) $$logit\left(\mathrm{Pr}(M=1|X,Z\right)=i_{M}+aX+d_{2}Z$$

(3) $$logit\left(\mathrm{Pr}(Y=1|X,M,Z\right)=i_{y3}+c\prime X+bM+hXM+d_{3}Z$$

Where *i*_*y1*_*, i*_*M*_*, and i*_*y3*_ are the intercept terms in the equations, *c* is the total effect of the exposure on the outcome, *c′* is the direct effect of the exposure on the outcome, *a* is the effect of the exposure on the mediator, *b* is the effect of the mediator on the outcome adjusted for the exposure, and *h* represents the effect of the exposure-mediator interaction on the outcome. *Z* represents the vector of confounders considered for each of the three models.

These equations also hold when the outcome is non-rare, and log-binomial models are fit to the data instead of logistic models, as in this study.^35^ In the absence of an exposure-mediator interaction, the coefficient *h* becomes zero, and the Natural Direct Effect (NDE), Natural Indirect Effect (NIE), and Total Effect (TE) are obtained using the coefficients from equations (2), (3)) as follows on the risk ratio scale as follows^34^^,^^36^:

(4) $$NDE_{RR}=\exp\left(c\prime \right)$$

(5) $$NIE_{RR}=\frac{\left(1+\exp(i_{M})(1+\exp(b+i_{M}+a\right)}{\left(1+\exp\left(i_{M}+a\right)\right)\left(1+\exp\left(b+i_{M}\right)\right)}$$

(6) $$TE_{RR}=NDE_{RR}*NIE_{RR}$$

We performed two sets of mediation analyses—one for each exposure. In the first mediation analysis (mediation-model-A), we considered *women's perceptions of health system capacities* as the exposure (*X*_*1*_), and in the second mediation analysis (mediation-model-B), we considered the *increased burden of domestic care* to be the exposure (*X*_*2*_). Both (mediation-model-A) and (mediation-model-B) considered *delayed healthcare seeking* as the mediator and *non-receipt of healthcare* as the outcome of interest.

For both the mediation analyses, we first tested for an interaction effect between the exposure and the mediator and, in both cases, found the coefficient of the interaction term to be non-significant. We then developed two sets of regression equations for each exposure, similar to equations (2), (3) above but with a log-link function instead of a logistic regression. We used the coefficients obtained from these models to calculate the NDE, NIE, and TE on the risk ratio scale. For both these models, we computed robust confidence intervals around the NDE and performed bootstrapping with 5000 iterations to estimate 95% Confidence Intervals around the NIE and TE. Finally, we estimated the proportion of the total effect mediated by the indirect effect for both the models using the following formula^36^:

(7) $$Proportion\;mediated=\frac{NDE_{RR}*\left(NIE_{RR}-1\right)}{\left(NDE_{RR}*\;NIE_{RR}-1\right)}$$

We performed all our analyses using R (version 4.1.3).

### Role of the funding source

The donor providing funding for this work had no involvement with the study design; collection, analysis, and interpretation of data; writing of the report, or decision to submit the paper for publication. The raw data was assessed by MG and data analysis was undertaken by AKD. AR made the decision to submit this paper for publication.

## Results

Participants (N = 1021) were married women with a mean age of 24 years (mean = 23.97, Std. Dev. = 2.98) and, on average, had completed 11 years of education (mean = 11.43, Std. Dev. = 3.15). Most were Hindu (92.36%) and belonged to the general caste category (70.32%); 84.52% had children. Only 5.09% reported that a member of their household showed symptoms of COVID-19 or tested positive for the virus in the past 30 days. More than one in ten women (12.34%) reported that they were concerned that healthcare was being directed only to address COVID-19. About two-thirds of the respondents (66.21%) reported that their domestic responsibilities increased for at least one activity, while the remaining 33.79% reported that their domestic responsibilities did not increase during the pandemic for any of the four activities (Table 1).

**Table-1:** Descriptive statistics for socio-demographic variables and covariates for total sample (N = 1021).

		Total Sample (N = 1021) n (%) or mean (std. dev.)	Did not need Healthcare for Self of Child (N = 691) n (%) or mean (std. dev.)	Needed Healthcare for Self or Child (N = 330) n (%) or mean (std. dev.)
Age (in years)		23.97 (2.98)	24.14 (3.01)	23.60 (2.86)
Years of education		11.43 (3.15)	11.36 (3.17)	11.58 (3.10)
Religion	Hindu	943 (92.36%)	636 (92.04%)	307 (93.03%)
	Non-Hindu	78 (7.64%)	55 (7.96%)	23 (6.97%)
Caste	Scheduled Caste	47 (4.60%)	31 (4.49%)	16 (4.85%)
	Scheduled Tribe	36 (3.53%)	24 (3.47%)	12 (3.64%)
	Other Backwards Class (OBC)^a^	220 (21.55%)	136 (19.68%)	84 (25.45%)
	General	718 (70.32%)	500 (72.36%)	218 (66.06%)
Parity	No child	158 (15.48%)	103 (14.91%)	55 (16.67%)
	1 child	549 (53.77%)	352 (50.94%)	197 (59.70%)
	2 or more children	314 (30.75%)	236 (34.15%)	78 (23.64%)
Any household member tested positive for COVID or showed symptoms of COVID	No	969 (94.91%)	659 (95.37%)	310 (93.94%)
	Yes	52 (5.09%)	32 (4.63%)	20 (6.06%)
Average monthly household income (in Rs.)		24,399.61 (42,337.71)	24,342.98 (38,266.65)	24518.18 (49,860.97)
Faced economic hardships during the pandemic	No	449 (44.98%)	322 (46.60%)	127 (38.48%)
	Yes	572 (56.02%)	369 (53.40%)	203 (61.52%)
Concerned that healthcare is only being directed to COVID-19 and not to other concerns (Exposure-1)	No	895 (87.66%)	624 (90.30%)	271 (82.12%)
	Yes	126 (12.34%)	67 (9.70%)	59 (17.88%)
Burden of domestic responsibilities increased during the pandemic (Exposure-2)	No	345 (33.79%)	258 (37.34%)	87 (26.36%)
	Yes -for 1 responsibility	219 (21.45%)	134 (19.39%)	85 (25.76%)
	Yes -for 2 responsibilities	169 (16.55%)	117 (16.93%)	52 (15.76%)
	Yes -for 3 responsibilities	169 (16.55%)	105 (15.20%)	64 (19.39%)
	Yes -for 4 responsibilities	119 (11.66%)	77 (11.14%)	42 (12.73%)
Did not receive healthcare for self or child when needed (Primary Outcome)	No			213 (64.55%)
	Yes			117 (35.45%)
Delayed seeking healthcare for self or child (Secondary Outcome)	No			249 (75.45%)
	Yes			81 (24.55%)

a This terminology represents that of the Government of India data collection process.

One-third of the respondents (32.32%) reported needing healthcare for themselves or their children during the pandemic. Among those who needed care, a quarter (24.55%) reported that they delayed seeking the care they needed due to the COVID-19 pandemic, and more than one-third (35.45%) reported not receiving healthcare for themselves or their children when needed during the pandemic. Those in need of healthcare were more likely than those not in need of care to be OBC (25.45% vs. 19.68%), report actual or suspected COVID-19 in the household (6.06% vs. 4.63%), report pandemic-related economic hardship (61.52% vs. 53.40%), and were concerned about the compromised health system capacities due to the pandemic (17.88% vs. 9.70%). They were also more likely to have one child (59.70% vs. 50.94%), though less likely to report having two or more children (23.64% vs. 34.15%) (Table 1).

### Regression models

Our adjusted model to predict non-receipt of healthcare (Model 1) found that women who were concerned that healthcare was focused solely on COVID were 1.7 times more at risk of not receiving healthcare when needed as compared to women who were not concerned about healthcare being diverted solely to COVID (Adjusted Risk Ratio [ARR] = 1.69, 95% CI = 1.27, 2.25). This model also found that an increased burden of household responsibilities was associated with non-receipt of healthcare. Women who reported an increased burden for two (ARR = 1.79, 95% CI = 1.06, 3.02), three (ARR = 2.15, 95% CI = 1.32, 3.49) or four (ARR = 2.53, 95% CI = 1.54, 4.16) household responsibilities were all more at risk of not receiving healthcare when needed as compared to women whose domestic responsibilities had not increased during the pandemic (Table 2).

**Table 2:** Adjusted Risk Ratio for the association of a) women's perception of healthcare capacity and b) burden of domestic care on not receiving healthcare (N = 330) [Model 1].

		Adjusted Risk Ratio	Robust 95% LCI	Robust 95% UCI
Concerned that healthcare is only being directed to COVID-19 and not to other concerns (Exposure-1)	No	Ref	–	–
	Yes	1.69	1.27	2.25
Burden of domestic responsibilities increased during the pandemic (Exposure-2)	No	Ref	–	–
	Yes -for 1 responsibility	1.12	0.64	1.94
	Yes -for 2 responsibilities	1.79	1.06	3.02
	Yes -for 3 responsibilities	2.15	1.32	3.49
	Yes -for 4 responsibilities	2.53	1.54	4.16

Model adjusted for women's age, education, parity, and any COVID-19 infections.

Our second model (Model 2), which was like Model 1 but additionally adjusted for delayed healthcare seeking, indicated that delayed healthcare seeking was significantly associated with non-receipt of care (ARR = 2.48, 95% CI = 1.90, 3.23). This model also showed that the coefficients for the two exposures were substantially reduced as compared to Model 1, indicating partial mediation due to delayed healthcare seeking (Table 3).

**Table 3:** Adjusted Risk Ratio for the associations in Model 1 additionally adjusted for delayed healthcare seeking (N = 330) [Model 2].

		Adjusted Risk Ratio	Robust 95% LCI	Robust 95% UCI
Concerned that healthcare is only being directed to COVID-19 and not to other concerns (Exposure-1)	No	Ref	–	–
	Yes	1.49	1.14	1.95
Burden of domestic responsibilities increased during the pandemic (Exposure-2)	No	Ref	–	–
	Yes -for 1 responsibility	1.14	0.68	1.94
	Yes -for 2 responsibilities	1.77	1.07	2.92
	Yes -for 3 responsibilities	2.22	1.39	3.55
	Yes -for 4 responsibilities	2.08	1.31	3.31
Delayed healthcare seeking for self or child due to COVID-19	No	Ref	–	–
	Yes	2.48	1.90	3.23

Model adjusted for women's age, education, parity, and any COVID-19 infections.

Our adjusted model to predict delayed healthcare seeking (Model 3) found a significant positive association for women who reported an increased burden for four domestic responsibilities (ARR = 1.84, 95% CI = 1.05, 3.21) but not for increased burden in three or fewer domestic responsibilities or for the concern about health system variable (exposure 1; ARR = 1.36, 95% CI = 0.88, 2.11). Though the latter (exposure 1) variable did suggest a trend in the hypothesised direction (Table 4).

**Table 4:** Adjusted Risk Ratio for the association of a) women's perception of healthcare capacity and b) burden of domestic care on delayed healthcare seeking (N = 330) [Model 3].

		Adjusted Risk Ratio	Robust 95% LCI	Robust 95% UCI
Concerned that healthcare is only being directed to COVID-19 and not to other concerns (Exposure-1)	No	Ref	–	–
	Yes	1.36	0.88	2.11
Burden of domestic responsibilities increased during the pandemic (Exposure-2)	No	Ref	–	–
	Yes -for 1 responsibility	1.01	0.57	1.79
	Yes -for 2 responsibilities	1.09	0.58	2.04
	Yes -for 3 responsibilities	0.89	0.47	1.65
	Yes -for 4 responsibilities	1.84	1.05	3.21

Model adjusted for women's age, education, parity, and any COVID-19 infections.

### Mediation models

Finally, our mediation models suggested that delayed healthcare seeking significantly mediated the association between the increased burden of domestic responsibilities and non-receipt of healthcare but not the association between concerns about the health system and non-receipt of healthcare.

**Fig. 3:**
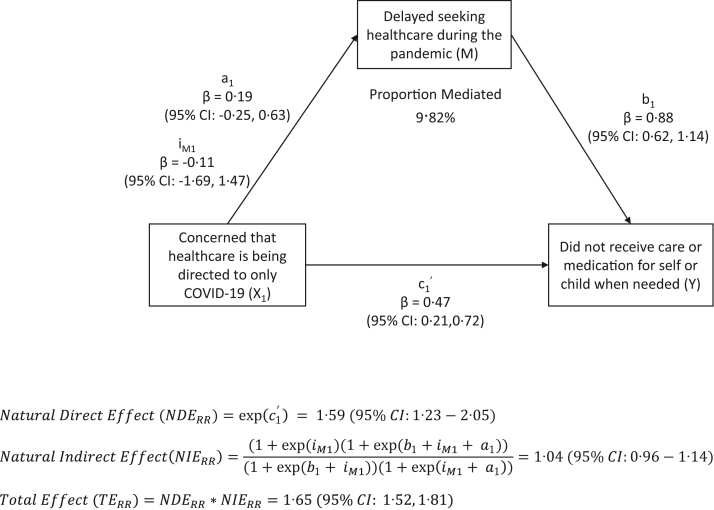
**Results from mediation-model-A with women's concern that healthcare is being directed only to COVID-19 as the exposure.** a_1_ is the coefficient for the exposure (X_1_) in the regression model with the Mediator (M) as the outcome. i_M1_ is the coefficient for the intercept in the regression model with the Mediator (M) as the outcome and the exposure (X_1_) as the predictor. b_1_ is the coefficient for the mediator (M) in the regression model with Y as the outcome, M as the exposure, while adjusting for (X_1_). c_1_^’^ is the coefficient for the exposure (X_1_) in the regression model with Y as the outcome and (X_1_) as the exposure, while adjusting for the mediator M.

**Fig. 4:**
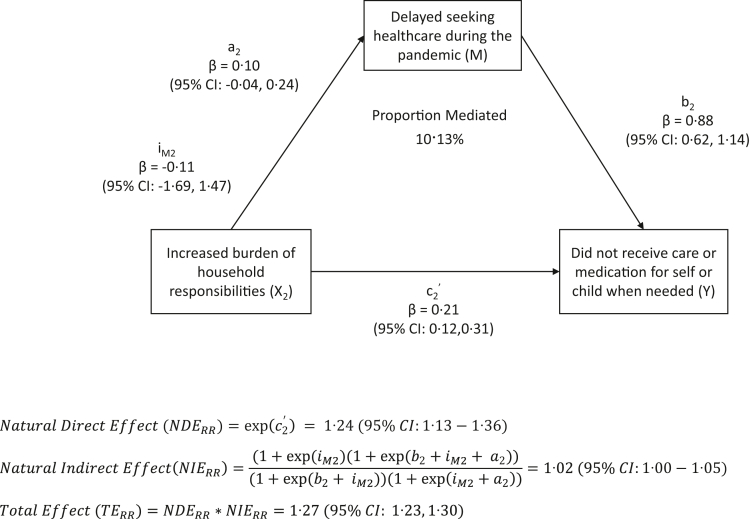
**Results from mediation-model-B with increased burden of household responsibilities as the exposure.** a_2_ is the coefficient for the exposure (X_2_) in the regression model with the Mediator (M) as the outcome. i_M2_ is the coefficient for the intercept in the regression model with the Mediator (M) as the outcome and the exposure (X_2_) as the predictor. B_2_ is the coefficient for the mediator (M) in the regression model with Y as the outcome, M as the exposure, while adjusting for (X_2_). c_2_^’^ is the coefficient for the exposure (X_2_) in the regression model with Y as the outcome and (X_2_) as the exposure, while adjusting for the mediator M.

The total effect of health system perceptions on non-receipt of healthcare decomposed into a Natural Direct Effect of 1.59 (95% CI = 1.23, 2.05) and a Natural Indirect Effect of 1.04 (95% CI = 0.96, 1.14) via delayed healthcare seeking. Delayed healthcare seeking mediated 9.82% of the total effect of health system perception on non-receipt of healthcare (Fig. 3). The total effect of increased burden of domestic responsibilities and non-receipt of healthcare decomposed into a Natural Direct Effect of 1.24 on the risk ratio scale (95% CI = 1.13, 1.36) and a Natural Indirect Effect of 1.02 (95% CI = 1.00, 1.05) via delayed healthcare seeking. Delayed healthcare seeking mediated 10.13% of the total effect of increased domestic care responsibilities and non-receipt of healthcare (Fig. 4).

## Discussion

In this study, we explored two potential predictors of women's non-receipt of needed health care under the pandemic for themselves or their children: 1) women's concerns that the health system is only responding to COVID cases and 2) increase in the burden of domestic responsibility under the pandemic. Importantly the study is set in the context of a COVID-19 hotspot in rural India, after the first wave and just before the second and more severe wave that incapacitated health systems.^2^ We found that more than one in ten women were already concerned that the system was not responding to or providing care beyond that for COVID-19 infection. We also found that both apprehensions about health system response and increased domestic labour were associated with women's pursuit and use of health care for themselves and their children. These findings from a community-based sample correspond with prior research with a clinical sample from India and globally.^16^, ^17^, ^18^ These findings highlight the importance of improving the perceptions about health systems' capacities and services offered, especially during times of crises, along with broader health service strengthening in India. They also highlight potential adverse health consequences of the well-documented increase in domestic labour burdens under the pandemic.^28^^,^^29^ More gender-equal distribution of labour in households can help address this concern.

Importantly, our findings also document that delay in healthcare seeking mediates our observed associations between our exposure variables—concerns that healthcare is focused on COVID and increased burden of domestic responsibilities, with our primary outcome—healthcare utilisation for needed care. Findings indicate that the effect of health system perceptions on reduced healthcare utilisation can be improved by up to 10 percent if we disable the pathway between concerns about health system capacity to provide care and delayed care-seeking. Similarly, healthcare utilisation can be increased by up to 10 percent if the pathway between increased domestic care responsibilities and delayed healthcare seeking is disabled.^37^ Prior research from other country contexts highlights that a lack of faith in health systems yields delayed care and unmet healthcare needs, particularly for socially marginalised groups and those with lesser geographic access to care,^37^ and these concerns have only been exacerbated under the COVID-19 pandemic.^38^ This research highlights that the issues also held true in rural India and were a concern even before the second wave of the pandemic when systems were so severely incapacitated.

Our findings suggest that there is an urgent need to address women's perceptions of health-system capacities during times of crisis. Strategies to strengthen healthcare provision through short-term solutions that allocate resources to under-staffed regions and long-term solutions such as policies around retention of healthcare workers in remote and rural areas can be helpful.^39^ In addition, outreach strategies at the community level via frontline health workers and media campaigns targeted at women and mothers can also be implemented to address their concerns related to capacities of the health system during crises. The second major issue that needs to be addressed is delayed healthcare-seeking behaviour. This can be achieved via rapid training of healthcare providers and community-level frontline health workers to include messages around avoiding delays in seeking healthcare when they meet their clients. Trained community level health workers can then include messages around not delaying healthcare needs to women during their counselling visits in communities. These are particularly important given the strain that COVID-19 caused the health system and given that India is actively working to strengthen its health system.

The study has a few limitations. First, the study uses cross-sectional data, and as a result, causality cannot be established. The study also relies solely on self-report data and did not collect any clinical data or medical records. This makes the study prone to social-desirability biases and limits our ability to confirm service utilisation. Secondly, we identified women and children who needed care during the pandemic via a single-item question. This precluded us from disentangling whether the mother or the child needed care and precluded us from undertaking analyses specific to women or children. Another important limitation of the study is the item used to capture delayed healthcare seeking asks women if they delayed or did not pursue healthcare when needed, a double-barrelled question. The item conflates delay in healthcare seeking and lack of pursuit of care. Nonetheless, the item is distinct from the outcome “non-receipt of care when needed” because care could still occur despite lack of pursuit of care in cases where other family members or community health workers intervened and directed the individual to care. However, results from the mediation analyses should be interpreted cautiously.

Furthermore, we did not collect information on the detailed reasons for non-availability of care other than the ones included in the study. This limits us in diving deeper into understanding the underlying reasons behind non-receipt of care. We also recognise limitations to the generalisability of the findings of this study. Our sample was drawn from a parent study that included married women aged 18–30 years, and thus our findings are generalisable only to that specific age range. Further, data used for the study was collected in a rapidly changing environment due to the evolving nature of the COVID-19 pandemic. This is a challenge because such changes can lead to changes in women's perceptions of the health system. However, the associations highlighted in our study between perceptions, delayed care-seeking, and healthcare utilisation makes an important contribution to expanding our current understanding of these pathways. Such an understanding can be extremely useful for taking preventive measures in case of upcoming waves or other crises that disrupt the delivery of healthcare services in India again.

In addition to the catastrophic morbidity and mortality affected by COVID-19, the pandemic also disrupted the health system and severely affected the provision of reproductive, maternal, and child health services. The pandemic, especially during its peaks, put significant burdens on the health system, which often led to the reallocation of resources from other healthcare services to COVID-19. Consequently, in this study, we find that these disruptions in health service delivery led women to have unfavourable perceptions about the capacities of the health system to cater to maternal and child healthcare services. We also find that these perceptions are associated with delayed healthcare seeking and non-receipt of care during the pandemic. These findings suggest an immediate effect on healthcare utilisation in India, which, if left unaddressed, can also lead to long-term impacts on maternal and child health outcomes. Therefore, it is critical to address these challenges rapidly, considering the threat of other COVID-19 waves continues to loom and new variants of the virus that can stress the health system again.

## Contributors

AKD was responsible for conceptualisation, Formal analysis, Writing-original draft, Writing-reviewing & editing. NB was responsible for conceptualisation, Writing-original draft, Writing-reviewing & editing. NR was responsible for investigation, Project administration, Resources, Supervision, Writing-reviewing & editing. MG was responsible for investigation, Project administration, Resources, Supervision, Data curation, Writing-reviewing & editing. SC was responsible for conceptualisation, Writing-original draft, Writing-reviewing & editing. AR was responsible for conceptualisation, funding acquisition, writing-original draft, writing-reviewing & editing. The raw data was assessed by MG and data analysis was undertaken by AKD. MG and AKD verified the data.

## Data sharing statement

The data that support the findings of this study after deidentification are available from the corresponding author upon reasonable request. Investigators whose proposed use of the data has been approved by an independent review committee (“learned intermediary”) identified for this purpose can access the data.

## Declaration of interests

All authors declare that they have no conflicts of interest.

## Appendix A Supplementary data

STROBE_checklist
